# Comparison of stem/progenitor cell number and transcriptomic profile in the mammary tissue of dairy and beef breed heifers

**DOI:** 10.1007/s13353-014-0213-1

**Published:** 2014-04-20

**Authors:** Ewa Osińska, Zofia Wicik, Michał M. Godlewski, Karol Pawłowski, Alicja Majewska, Joanna Mucha, Małgorzata Gajewska, Tomasz Motyl

**Affiliations:** 1 Department of Physiological Sciences, Faculty of Veterinary Medicine, Warsaw University of Life Sciences—SGGW, Nowoursynowska 159, 02-776 Warsaw, Poland; 2 Department of Human Epigenetics, Mossakowski Medical Research Centre, Polish Academy of Sciences, Pawińskiego 5, 02-106 Warsaw, Poland; 3 Department of Large Animal Diseases with Clinic, Faculty of Veterinary Medicine, Warsaw University of Life Sciences—SGGW, Nowoursynowska 100, 02-797 Warsaw, Poland

**Keywords:** Stem/progenitor cells, Transcriptomics, Mammary gland, Dairy and beef heifers

## Abstract

Bovine mammary stem cells (MaSC) are a source of ductal and lobulo-alveolar tissue during the development of the mammary gland and its remodeling in repeating lactation cycles. We hypothesize that the number of MaSC, their molecular properties, and interactions with their niche may be essential in order to determine the mammogenic potential in heifers. To verify this hypothesis, we compared the number of MaSC and the transcriptomic profile in the mammary tissue of 20-month-old, non-pregnant dairy (Holstein-Friesian, HF) and beef (Limousin, LM) heifers. For the identification and quantification of putative stem/progenitor cells in mammary tissue sections, scanning cytometry was used with a combination of MaSC molecular markers: stem cell antigen-1 (Sca-1) and fibronectin type III domain containing 3B (FNDC3B) protein. Cytometric analysis revealed a significantly higher number of Sca-1^pos^FNDC3B^pos^ cells in HF (2.94 ± 0.35 %) than in LM (1.72 ± 0.20 %) heifers. In HF heifers, a higher expression of intramammary hormones, growth factors, cytokines, chemokines, and transcription regulators was observed. The model of mammary microenvironment favorable for MaSC was associated with the regulation of genes involved in MaSC maintenance, self-renewal, proliferation, migration, differentiation, mammary tissue remodeling, angiogenesis, regulation of adipocyte differentiation, lipid metabolism, and steroid and insulin signaling. In conclusion, the mammogenic potential in postpubertal dairy heifers is facilitated by a higher number of MaSC and up-regulation of mammary auto- and paracrine factors representing the MaSC niche.

## Introduction

The bovine mammary gland is a unique organ with regard to its frequently repeating cycles of growth and involution throughout the life of an animal. Although the general processes controlling mammogenesis have been extensively studied, the knowledge on the role of stem cells and their renewal during mammary gland development is still insufficient. Mammary stem cells (MaSC) are defined as cells that can generate the ductal and lobular components of the mammary epithelial tree, complete with all the cell types of the mammary epithelium, as well as having the ability to self-renew (Stingl 2009). Stem cells allow the mammary epithelium to expand intensively during puberty and pregnancy, preparing the gland for milk production and secretion during lactation (Daniel and Smith 1999). To confirm the presence of MaSC, many in vitro and in vivo studies on rodents were conducted with the use of transplantation experiments, electron microscopy, functional techniques, flow cytometry, scanning cytometry, microarrays, and mammosphere cultures. Unfortunately, until now, a universal molecular stem cell marker for the identification of these cells has not been found. The most successful approach used to identify mouse MaSC has been based on a combination of surface markers: CD24 (heat-stable antigen), CD29 (*β*1 integrin), CD49f (*α*6 integrin), CD61 (*β*3 integrin), and Sca-1 (stem cell antigen-1) (Shackleton et al. 2006; Stingl et al. 2006; Han et al. 2006). In the mouse, CD24 is a pan-epithelial marker that functions as a crude epithelial–stromal discriminator (Sleeman et al. 2006; Stingl 2009). However, in the human mammary gland, CD24 is a luminal cell marker with a similar distribution to the luminal cell-specific glycoprotein MUC1. Thus, the most useful combination of molecular markers for isolating human MaSC comprised the epithelial cell adhesion molecule [EpCAM; also known as epithelial specific antigen (ESA) and CD326], CD49f, and, to a lesser degree, the luminal cell-specific glycoprotein MUC1 (Eirew et al. 2008; Villadsen et al. 2007). The cells expressing the above-mentioned markers were shown to form mammary repopulating units (MRU), which, when transplanted into cleared mammary fat pads of recipient mice, were able to repopulate the fat pad and recreate the structure of the mammary gland (Stingl et al. 2006; Eirew et al. 2008; Villadsen et al. 2007). Although a cleared fat pad technique was also described for ruminant species (Hovey et al. 1999), utilization of the technique has been very limited due to inherent differences between the composition of stroma in rodents and ruminants. Mouse stroma is composed mainly of adipocytes, whereas stromal tissue of the bovine mammary gland is fibrous (Sheffield 1988; Ellis et al. 2012). Furthermore, the global structure of the mammary gland differs significantly between rodents and ruminants. Murine mammary epithelium is a tree-like system of ducts terminated by numerous alveoli, whereas in ruminants, mammary alveoli and converging ducts form terminal duct lobular units (TDLU), which are gathered in a form of lobes.

In the attempt to define the bovine MaSC population, some promising results were obtained from the experiments based on the ability of these cells to retain the bromodeoxyuridine (BrdU) label for an extended period of time (Capuco 2007; Capuco et al. 2009). Stem cells were demonstrated to retain labeled DNA because of their selective segregation of template DNA strands during mitosis. These cells, described as label-retaining epithelial cells (LRECs), were detected immunohistochemically and quantified (Capuco 2007). The studies showed that the size of the bovine LREC population averaged 0.4 %, but could be doubled by xanthosine treatment, due to xanthosine-evoked suppression of the p53 function, which resulted in the promotion of asymmetric stem cell division (Capuco et al. 2009).

In a recently published study, Rauner and Barash (2012) utilized fluorescence-activated cell sorting (FACS) to distinguish and characterize four populations of cells within the bovine mammary gland on the basis of the expression of CD24 and CD49f surface markers. The authors have demonstrated that putative stem cells (puStm) were CD24^med^CD49f ^pos^, had basal localization, and preserved the ability to generate organized clones with duct-like cell alignment in culture. The populations of putative progenitors (puPgt: CD24^high^CD49f^neg^) and basal cells (CD24^neg^CD49f^pos^) were located downstream of puStm in the hierarchy, with puPgt having bi-potent characteristics, whereas the basal population complemented the puStm population to form the basal compartment. Finally, luminal cells (Lum) were CD24^med^CD49f^neg^ and complemented the puPtg cells in comprising the luminal compartment, and localized in the lower boundary of the luminal lineage.

Our group previously utilized stem cell antigen-1 (Sca-1) in the search for putative stem/progenitor cells in the bovine mammary gland (Motyl et al. 2011). The results demonstrated that bovine mammary epithelial cells expressing Sca-1 comprised about 2 % of the total cell number in the mammary tissue. Sca-1^pos^ cells were ERα-negative, indicating a possibility that Sca-1^pos^ cells form a population which localizes in the mammary stem/progenitor niches, and is important for the renewal of the bovine mammary gland during development and tissue regeneration. Transcriptomic analysis of the Sca-1^pos^ cell population in comparison with Sca-1^neg^ cells showed that the differentially expressed genes were involved in biological processes, such as signal transduction, development, protein metabolism and protein modifications, cell structure, motility, immunity, and defense (Motyl et al. 2011).

It is very likely that the number and unique morphological and molecular features of stem cells predispose the mammary gland to a certain type and dynamics of growth. Probably, numerous signals from the extracellular matrix (ECM) also affect the pattern of growth of the mammary gland. In fact, local tissue microenvironment composed of progenitor cells, basement membrane, ECM, stromal cells, and soluble factors, such as hormones and growth factors, create a functional signaling niche that directs cellular activity via direct contact or paracrine signaling (Bussard and Smith 2011). Thus, the number of stem cells, as well as the composition of the microenvironment, may determine the rate of development of the mammary gland. It is generally accepted that the intensity of proliferation and the productivity of the mammary gland in beef breeds and dairy breeds differs significantly (Keys et al. 1989; Akers et al. 2006). Up to now, no research has been carried out showing the relationship between the bovine mammary stem/progenitor cell number and interbreed differences in the intensity of mammary tissue outgrowth and development. The present study was undertaken to fulfill this gap by the quantification of stem/progenitor cells in the mammary tissue of non-pregnant 20-month-old Holstein-Friesian (HF) heifers—a typical dairy breed—and Limousin (LM) heifers—a typical beef breed. Moreover, a comparison of transcriptomic profiles of mammary tissue was performed in order to identify genes which could facilitate the formation of favorable tissue environment for self-renewal and differentiation of stem/progenitor cells. It seems essential to determine how the number of stem/progenitor cells and the influence of intramammary auto- and paracrine factors may impact growth, morphology, and productivity of the mammary gland of cattle with different phenotypes. Exploration of these issues may allow the use of the specific features of stem cells to control the development of the mammary gland, leading to a higher productivity of dairy cows, shortening the recovery time of the gland, controlling its defense mechanisms, and maybe even manipulating the milk composition.

## Materials and methods

### Tissue sampling

Mammary tissue was obtained at a slaughterhouse from the udders of individual 20-month-old non-pregnant HF heifers (*n* = 10) and LM heifers (*n* = 10), free of clinical signs of mastitis. Udders were removed and mammary tissue was excised from the well-distinguished parenchymal region near the border with the mammary stroma. The samples were collected and immediately fixed, using different procedures, for scanning cytometry and microarray assay.

### Immunofluorescent staining of tissue sections for scanning cytometry

For immunofluorescent staining, samples were fixed in 4 % phosphate-buffered formalin and, after 48 h, stored in 70 % ethanol (POCH S.A., Gliwice, Poland) until further processing. Tissues were dehydrated and paraffin-embedded, according to standard histological technique. The paraffin blocks were cut into sections (5 μm), which were mounted on silanized microscope slides. Next, slides were deparaffined in xylene and hydrated in a graded series of ethanol to phosphate-buffered saline (PBS). For antigen retrieval, tissue sections were heated in a microwave (650 W) in 400 ml of 10 mM citrate buffer (ph 6.0), according to Capuco (2007). Next, they were rinsed with PBS.

For scanning cytometry, tissue sections were labeled with fibronectin type III domain containing 3B (FNDC3B) rabbit polyclonal antibody (Santa Cruz Biotechnology), followed by incubation with secondary anti-rabbit Alexa Fluor 647-conjugated antibody (Life Technologies, USA), and with mouse anti-Sca-1-FITC-conjugated antibody (BD Pharmingen, USA). Nuclei were counterstained with Hoechst 33342. Scanning cytometry analysis was performed using an Olympus Scan^R screening station (Olympus Polska, Sp. z o. o., Warsaw, Poland), and combined analysis software (SCAN^R Analysis version 1.3.03). The results were statistically evaluated using Microsoft Excel 2003 software (Microsoft Corporation, Redmond, WA, USA) to calculate the mean number of Sca-1^pos^FNDC3B^pos^ cells ± standard deviation (SD). Statistical significance was calculated by one-way analysis of variance (ANOVA) comparing the number of Sca-1^pos^FNDC3B^pos^ cells in mammary tissue samples from LM and HF heifers (GraphPad Prism software version 5.00). A *p*-value ≤ 0.05 was regarded as significant.

### Microarray analysis

For microarrays, mammary tissue samples were snap-frozen in RNAlater (Sigma Aldrich, Poland) and stored at −80 °C. The total RNA was isolated from 50 mg of each tissue sample using a Total RNA kit (A&A Biotechnology, Poland), according to the manufacturer’s protocol. Isolated RNA samples were dissolved in RNase-free water. The quantity of RNA was measured using NanoDrop (NanoDrop Technologies, USA). Next, RNA samples were treated with DNase-I to eliminate the possibility of DNA contamination, and subsequently purified using the RNeasy MinElute Cleanup Kit (Qiagen, Germany). Finally, RNA was analyzed using a BioAnalyzer (Agilent, USA) to measure the final RNA quality and integrity. Samples with RIN between 7 and 8.5 were used for further analysis. Amplification and labeling of target RNA was done using the Quick Amp Labeling Kit (Agilent, USA) in order to generate complementary RNA (cRNA) for oligo microarrays used in gene expression profiling and other downstream analyses. Prior the labeling procedure, equal amounts of RNA (500 ng) from each mammary tissue were pooled (HF pool from 10 samples and LM pool from 10 samples). We chose to use the pooling of RNA because the number of animals in each compared group was relatively small (*n* = 10) and this method allowed us to decrease the variability between animals within a group (HF and LM). The gene expression of the HF heifers was compared against the gene expression of the LM heifers’ mammary tissue. Samples were examined in four repetitions (two dye-swaps to eliminate the effect of label factor). Taking the average of all labeled arrays, the dye effect on any particular gene was cancelled. The hybridization was performed with bovine-specific gene expression Agilent microarrays (4x44K format, no. of probes = 43603) using the Gene Expression Hybridization Kit (Agilent, USA), according to the manufacturer’s protocol. The results obtained constitute only a comparison of the “average” expression in one group versus another (HF vs. LM heifers). Although the design used in the analysis was based on four technical repetitions of the two pooled samples, the results obtained were used only for direct comparison, and were not correlated with any individual data, thus, they are acceptable from the methodological point of view (Kendziorski et al. 2003).

### Signal detection, quantification, and analysis

Acquisition and analysis of hybridization intensities were performed using a DNA microarray scanner (Agilent, USA). Then, the results were extracted using Agilent’s Feature Extraction Software with normalization and robust statistical analyses. Analysis of datasets was performed using GeneSpring software (Agilent, USA). Raw data were preprocessed to remove variability across and within array samples. To minimize non-biological variability across arrays, raw data were first log2 transformed. All data were filtered by flags present in all samples. The unpaired *t*-test with Benjamin–Hochberg false discovery rate (FDR) <5 % correction was applied (with *p*-value cut-off ≤0.05). For further analysis, we chose genes with significant changes in expressions of over 1.3-fold change. The area of the analyses covered in this publication has been deposited in the NCBI’s Gene Expression Omnibus and is accessible via GEO Series accession number GSE47816.

Gene functions were identified using the Gene Ontology (GO) annotation of genes in the Functional Annotation Chart, available through the online Database for Annotation, Visualization and Integrated Discovery (DAVID). DAVID was used to analyze gene sets significantly differing in expression between the mammary glands of HF and LM heifers. Analysis of signaling pathways was carried out using Single Experiment Analysis (SEA) exact in GeneSpring (Agilent, USA). The significance of the association between the dataset and the GO terms were measured using the ratio of the number of genes from the dataset that map to the pathway divided by the total number of genes in the test. A threshold of *p*-value ≤ 0.05 was used to indicate biological processes, molecular functions, and signaling pathways that are significantly represented by genes in an annotated gene list. To interpret the results, genes’ functions and interactions were analyzed based on the literature, and genes’ interactions networks were developed using Pathway Studio software (Ariadne Genomics, Inc.).

## Results

### Identification and quantification of mammary stem/progenitor cells

To identify putative mammary stem/progenitor cells, we applied double labeling with previously used anti-Sca-1-FITC-conjugated antibody (Motyl et al. 2011) and antibody against FNDC3B, which is considered a putative marker of mammary stem/progenitor cells. Previous studies have shown that FNDC3B can be used as a potential marker of LREC in the bovine mammary gland (Choudhary and Capuco 2012). Cytometric analysis of the number of cells expressing both FNDC3B and Sca-1 antigens revealed their significantly higher value in HF (2.94 ± 0.35 %) than in LM (1.72 ± 0.20 %) heifers. Their undifferentiated status was confirmed by the lack of ERα expression (data unpublished).

### Transcriptomic profiles of the mammary tissue of dairy and beef breed heifers

The transcriptomic background of a higher mammogenic potential in HF heifers was investigated by comparing the differences between gene expression profiles in the mammary tissue of HF and LM heifers. Fold change analysis followed by Student’s *t*-test and Benjamini–Hochberg FDR correction (*p*-value ≤ 0.05) identified 3,153 probes representing 1,987 genes that were significantly altered by at least 1.3-fold between the two examined breeds. The classification of genes according to their molecular function revealed four major groups of up-regulated genes in HF heifers: transcription regulator activity (53 genes), cytokine activity (17 genes), growth factor activity (12 genes), and chemokine activity (9 genes). Among genes with significant changes in expression, we selected those representing tissue hormones, growth factors, and cytokines, which expression was linked with the function of the mammary gland (Table 1). Special attention was focused on the expression of genes whose products could affect mammary stem/progenitor cells (Table 2). Among them were genes involved in cell maintenance, stem cell renewal, and stem cell development.

**Table 1:** Main growth factors, hormones, and cytokines up-regulated in the mammary tissue of Holstein-Friesian (HF) in comparison with Limousin (LM) heifers. Gene Ontology (GO) analysis was performed for genes that significantly expressed over a 1.3-fold change absolute (*FC*) with false discovery rate (FDR) corrected *p*-value cut-off ≤0.05

Gene symbol	Description	FC	*p*-Value	Possible function in the mammary gland
BTC	betacellulin [NM_173896]	2.6	0.024	EGF family member; promotion of growth and differentiation
CSF1	colony-stimulating factor 1 (macrophage) [Source:RefSeq peptide;Acc:NP_776451] [ENSBTAT00000000353]	1.4	0.011	Mammary gland development during pregnancy and lactation
CSF2	colony-stimulating factor 2 (granulocyte-macrophage) [NM_174027]	2.9	0.046	Control of proliferation, differentiation, and function of granulocytes and macrophages
CSF3	colony-stimulating factor 3 (granulocyte) [NM_174028]	7.1	0.008	Control of proliferation, differentiation, and function of granulocytes and macrophages
ESM1	endothelial cell-specific molecule 1 [NM_001098101]	4.8	0.026	Inhibition of leukocyte adhesion and migration through the endothelium
FGF18	fibroblast growth factor 18 [NM_001076007]	2.1	0.025	Promotion of proliferation, differentiation, and matrix production
FGF2	fibroblast growth factor 2 (basic) [NM_174056]	1.8	0.021	Stimulation of ductal outgrowth and differentiation; regulation of mammary stem/progenitor cells
GDF15	PREDICTED: growth/differentiation factor 15-like [XM_871003]	1.7	0.013	Regulation of growth and differentiation; anti-inflammatory effect
GRO1	chemokine (C-X-C motif) ligand 2 [NM_175700]	25.9	0.009	Expressed in macrophages; role in inflammatory response
LIF	leukemia inhibitory factor (cholinergic differentiation factor) [NM_173931]	12.7	0.011	STAT3-dependent self-renewal of stem cells and maintenance in their undifferentiated state; initiation of apoptosis; promotes long-term maintenance of embryonic stem cells by suppressing spontaneous differentiation
NGF	nerve growth factor (beta polypeptide) [NM_001099362]	2.1	0.049	Development of nerve fibers; mitogenic in breast cancer cells
NRG1	neuregulin 1 [NM_174128]	1.5	0.006	Stimulation of branching morphogenesis, lobulo-alveolar budding, and production of milk proteins
OSGIN1	oxidative stress induced growth inhibitor 1 [NM_001077129]	1.6	0.043	Regulation of proliferation differentiation of MECs; regulation of apoptosis
PTN	pleiotrophin [NM_173955]	1.4	0.027	Growth, migration, and invasion of MECs; inhibition of expression and differentiation of progenitor cells; inhibition of ductal branching and outgrowth
WISP2	WNT1 inducible signaling pathway protein 2 [NM_001102176]	1.9	0.011	Estrogen-dependent cell growth and differentiation regulator
WISP2	cDNA clone MGC:159680 IMAGE:8111916 [BC151629]	1.8	0.021	Estrogen-dependent cell growth and differentiation regulator
WISP3	PREDICTED: hypothetical LOC784564 [XM_001252834]	1.7	0.036	Inhibition of growth and angiogenesis in breast cancer; modulation of IGF signaling
RETN	resistin [NM_183362]	1.6	0.028	Inhibition of adipocyte differentiation; control of insulin-dependent glucose uptake in MECs
NTS	neurotensin [NM_173945]	3.1	0.021	Synaptic transmission In peptidergic nerve fibers; anti-apoptotic effect
GRP	gastrin-releasing peptide [NM_001101239]	1.8	0.011	Proliferation, differentiation, and hypertrophy of the mammary gland
EDN1	endothelin 1 (EDN1) [NM_181010]	1.9	0.026	Cell differentiation, proliferation, and migration; activation of anti-apoptotic signals; stimulation of angiogenesis
ADM	adrenomedullin [NM_173888]	1.6	0.009	Tissue survival; modulator of inflammatory processes, blood circulation, and vasoconstriction
ADIPOQ	adiponectin, C1Q, and collagen domain containing [NM_174742]	1.6	0.029	Complement activation; carbohydrate transport; signal transduction; cell adhesion; gluconeogenesis; fatty acid beta-oxidation; signal transduction; cell adhesion; cellular component morphogenesis; mesoderm development; skeletal system development; response to stimulus; gonadotropin-releasing hormone receptor pathway
ADIPOQ	adiponectin precursor (adipocyte, C1q, and collagen domain-containing protein) [source:UniProtKB/Swiss-Prot;Acc:Q3Y5Z3] [ENSBTAT00000026395]	1.5	0.038	Complement activation; carbohydrate transport; signal transduction; cell adhesion; gluconeogenesis; fatty acid beta-oxidation; signal transduction; cell adhesion; cellular component morphogenesis; mesoderm development; skeletal system development; response to stimulus; gonadotropin-releasing hormone receptor pathway
IL1A	interleukin 1, alpha [NM_174092]	7.2	0.021	Proinflammatory mediator
IL1B	interleukin 1, beta [NM_174093]	11.4	0.008	Proinflammatory mediator
IL34	interleukin 34 [NM_001100324]	1.4	0.026	Immune response: promotion of monocyte survival; differentiation of monocytes into immunosuppressive macrophages
IL6	interleukin 6 (interferon, beta 2) [NM_173923]	55.7	0.010	STAT3-dependent regulation of stem cells; apoptosis induction, triggering malignant features in mammospheres from stem cells; promotes breast cancer cell growth
IL8	interleukin 8 [NM_173925]	51.6	0.005	Early inflammatory response; local immune response; macrophage activation; promoter of angiogenesis
PF4	platelet factor 4 [NM_001101062]	2.6	0.037	Positive regulation of gene expression; stimulation of TNF production; regulation of angiogenesis; platelet activation; blond coagulation; immune response
TNF	tumor necrosis factor [NM_173966]	10.5	0.005	Multifunctional role In the regulation of growth and development: stimulation of ductal and lobular morphogenesis, stimulation of proliferation, differentiation (in the presence of EGF and lactogenic factors), inhibits casein gene expression; mobilization of innate and acquired immunity
FST	follistatin [NM_175801]	1.9	0.011	Regulation of bovine mammary branching morphogenesis and MEC differentiation; regulation of renewal and development of stem/progenitor cells
FAS	Fas (TNF receptor superfamily, member 6) [NM_174662]	1.6	0.040	Remodeling of mammary tissue; cell cycle; signal transduction
CXCR2	interleukin 8 receptor, beta [NM_174360]	20.2	0.017	Immune response; mammary stem cells migration

**Table 2:** Genes involved in the regulation of stem/progenitor cells in the mammary tissue of HF in comparison with LM heifers. GO analysis was performed for genes that significantly expressed over a 1.3-fold change absolute (*FC*) with threshold FDR corrected *p*-value cut-off ≤0.05

Gene symbol	Description	FC	*p*-Value	Possible function in the mammary gland
Stem cells maintenance				
LIF	leukemia inhibitory factor (cholinergic differentiation factor) [NM_173931]	12.7	0.011	Promotes long-term maintenance of embryonic stem cells by suppressing spontaneous differentiation
CDKN1A	cyclin-dependent kinase inhibitor 1A (p21, Cip1) [NM_001098958]	3.0	0.011	Human embryonic stem cells (hESCs) commonly describe the non-functional p53-p21 axis of the G1/S checkpoint pathway with subsequent relevance for cell cycle regulation and the DNA damage response (DDR)
POSTN	periostin, osteoblast-specific factor [NM_001040479]	2.2	0.013	Increase cancer stem cell maintenance, and blocking its function prevents metastasis; increases wnt signaling in cancer stem cells.
ID1	inhibitor of DNA binding 1, dominant negative helix-loop-helix protein [NM_001097568]	2.1	0.011	Increase cell motility, decrease stem cell maintenance
JAK2	O19064_PIG (O19064) JAK2, partial (47 %) [TC366796]	1.9	0.026	Regulates lactation and alveologenesis
FGF2	fibroblast growth factor 2 (basic) [NM_174056]	1.8	0.021	Promote differentiation of stem cells to mesodermal lineages; expressed in basal and luminal mammary epithelial cells
PPARG	peroxisome proliferator-activated receptor gamma [NM_181024]	1.6	0.011	Induces the proliferation of mammary cancer stem cells; therapeutic target in triple-negative breast cancers; increases asymmetric cell division (HSCs)
BIRC5	baculoviral IAP repeat containing 5 [NM_001001855]	−1.4	0.049	Regulator of Wnt target genes; gene expression is high during fetal development and in most tumors, yet low in adult tissues
ANGPT1	angiopoietin 1 [NM_001076797]	−1.4	0.036	Mediates reciprocal interactions between the endothelium and surrounding matrix and mesenchyme; inhibits endothelial permeability; blood vessel maturation and stability
EZH2	enhancer of zeste homolog 2 [NM_001193024]	−2.2	0.011	Overexpression in stem cell self-renewal; breast cancer progression
NEUROD1	neurogenic differentiation 1 [NM_001103288]	−8.5	0.008	Regulates expression of the insulin gene; chemosensitivity marker in estrogen receptor-negative breast cancer
ASCL2	achaete-scute complex homolog 2 [NM_001040607]	−2.4	0.007	Basic helix-loop-helix (bhlh) transcription factor, controls the fate of intestinal stem cells
TCF7L1	PREDICTED: transcription factor 7-like 1 (T-cell specific, HMG-box) [XM_593301]	−1.3	0.039	Activated by beta catenin, mediates the Wnt signaling pathway and is antagonized by the transforming growth factor beta signaling pathway; terminal differentiation of epidermal cells
LRP5	Low-density lipoprotein receptor-related protein 5 fragment [Source:UniProtKB/TrEMBL;Acc:Q1MW21] [ENSBTAT00000007756]	−2.4	0.044	The Wnt signaling receptor required for canonical Wnt activity; enriches stem cell activity; decreases expression of senescence-associated markers
Stem cells renewal				
IL8	interleukin 8 [NM_173925]	51.6	0.005	Chemokine produced by macrophages and other cell types, such as epithelial cells; synthetized by endothelial cells, which store IL-8 in their storage vesicles; potent promoter of angiogenesis; increases the formation of primary and secondary mammospheres
CEBPD	CCAAT/enhancer binding protein (C/EBP), delta [NM_174267]	2.6	0.011	bZIP transcription; immune and inflammatory responses; activation and/or differentiation of macrophages; regulates adipocyte differentiation
ADM	adrenomedullin [NM_173888]	1.6	0.009	Growth and cell fate regulatory factor for adult neural stem and progenitor cells; regulates the proliferation rate and the differentiation into neurons, astrocytes, and oligodendrocytes of stem/progenitor cells through the PI3K/Akt pathway; stimulates vasodilation and angiogenesis
HOXB4	homeobox B4 [NM_001078114]	1.6	0.010	Activates Myc and down-regulates in TNF-α and FGF signaling; stimulator of stem cells self-renewal
MYC	v-myc myelocytomatosis viral oncogene homolog (avian) [NM_001046074]	1.5	0.036	Increase stem cells self-renewal
BMI1	BMI1 polycomb ring finger oncogene (BMI1) [NM_001038072]	1.4	0.049	Regulates stem cells proliferation and differentiation of committed cells in mammary epithelium
MLL5	myeloid/lymphoid or mixed-lineage leukemia 5 (trithorax homolog, Drosophila) [NM_001082451]	1.3	0.033	Cell cycle progression; key regulator of hematopoiesis involved in terminal myeloid differentiation and in the regulation of hematopoietic stem cell (HSCs); stem cells self-renewal
FGFR2	*B. taurus* mRNA for FGF-receptor [Z68150]	−1.5	0.033	Promotes breast tumorigenicity through maintenance of breast tumor-initiating cells; potent mitogenic activity for a wide variety of epithelial cells; paracrine mediator of normal epithelial cell proliferation
SERPINF1	serpin peptidase inhibitor, clade F (alpha-2 antiplasmin, pigment epithelium derived factor), member 1 [NM_174140]	−1.6	0.012	Strongly inhibits angiogenesis
E2F1	PREDICTED: E2F transcription factor 1 [XM_615437]	−2.5	0.042	Transcription factor that regulates the expression of target genes whose products participate in DNA replication, mitotic check point, mitosis, DNA damage checkpoints, and DNA repair; regulator of proliferation; critical role in cell-cycle progression and the induction of apoptosis in response to DNA damage
STAT5A	signal transducer and activator of transcription 5A [NM_001012673]	−2.1	0.017	Regulates mammary alveologenesis; necessary and sufficient for the establishment of luminal progenitor cells; activated by prolactin, growth hormone, and EGF
SFRP2	secreted frizzled-related protein 2 [NM_001034393]	−1.7	0.040	Modulates Wnt signaling in endothelial cells; induces angiogenesis; regulator for adipose tissue-derived stem cells; induce cellular resistance to apoptosis in mammary tumors
Stem cell development				
IL6	interleukin 6 (interferon, beta 2) [NM_173923]	55.7	0.010	Migration, negative regulation of fat cell differentiation, positive regulation of cell proliferation, insulin signaling; inhibits secretion of aldosterone; promotes breast cancer cell growth
TAC1	tachykinin, precursor 1 [NM_174193]	8.4	0.017	Encodes peptides that target: nerve receptors, immune cells, stem cells, hematopoietic cells, and smooth muscle cells; function in vasodilatory responses; expression occurs in breast cancer and is directly proportional to the aggressiveness of the prognostic factor in breast cancer
NGF	nerve growth factor (beta polypeptide) [NM_001099362]	2.1	0.049	Extracellular ligand for the NTRK1 and NGFR receptors; activates cellular signaling cascades through those receptor tyrosine kinase to regulate neuronal proliferation, differentiation, and survival; can be targeted in breast cancer to inhibit tumor cell proliferation, survival, and metastasis
MYB	v-myb myeloblastosis viral oncogene homolog (avian) [NM_175050]	−1.3	0.027	Controls the proliferation and differentiation of hematopoietic stem and progenitor cells

The analysis of signaling pathways, which differed significantly between HF and LM, and could have a greater impact on mammary gland development and milk production, was performed using the GeneSpring SEA functional pathway analysis tool. The greatest differences between HF and LM were observed in the case of genes that are associated with adipogenesis signaling (*p* = 0.00E + 00; 39 genes), autophagy (*p* = 8.52E −09; 24 genes), estrogen metabolism (*p* = 3.18e −06; 8 genes), cell cycle (*p* = 6.34e −06; 19 genes), apoptosis (*p* = 1.02e −05; 17 genes), insulin signaling (*p* = 4.64e −04; 22 genes), EGFR1 signaling pathway (*p* = 7.05e −04; 24 genes), and ID signaling pathway (*p* = 9.38E −03; 9 genes).

On the basis of the functions of genes presented in Tables 1 and 2, and the available literature, we were able to predict and discuss the role of the identified genes in the mechanisms controlling stem/progenitor cells function in outgrowth and development of the mammary tissue.

## Discussion

There is a great deal of evidence showing the importance of pubertal mammary development as a determinant of heifers’ milk yield potential. Mammary growth during puberty is affected by endocrine regulation, mainly ovarian and somatotropic axis hormones, as well as nutrition, including feeding level and specific nutrients (Sejrsen 1994). The results of the present study revealed that, apart from central endocrine and nutritional mechanisms controlling mammogenesis, the intra-mammary potential of the stem/progenitor cells population may influence better mammary tissue outgrowth and development in dairy heifers. We were able to show a higher number of stem/progenitor cells and up-regulation of genes involved in the formation of favorable niche for the maintenance, renewal, proliferation, and differentiation of stem/progenitor cells in HF than LM heifers (Tables 1 and 2). The higher number of stem/progenitor cells, as well as the elevated expression of genes involved in the growth and differentiation of the glandular tissue, can make the postpubertal development of mammary tissue more advanced, and the gland better prepared for pregnancy and lactation in HF heifers.

### Estimation of the mammary stem/progenitor cell number in different breed heifers

Many potential molecular markers have been investigated in search of a universal stem cell marker, or a set of markers that would allow assessment of the number of stem cells within the mammary gland. The approach used in the identification and isolation of MaSC from murine and human mammary glands was based on a combination of several surface markers: CD24, CD29 (*β*1 integrin), and CD49f (*α*6 integrin) (Stingl et al. 2006; Shackleton et al. 2006; Eirew et al. 2008). Recently, Rauner and Barash (2012) used FACS to determine the expression of CD24 and CD49f in mammary epithelial cells (MECs) isolated from the bovine mammary gland. On the basis of the expression levels of both surface markers, the authors distinguished four different subpopulations of bovine mammary epithelial cells, of which the putative stem cells population (puStm) was characterized by moderate levels of CD24 and high levels of CD49f (CD24^med^CD49f^high^). The measurement of aldehyde dehydrogenase (ALDH) activity in bovine MECs was also used as a marker, allowing for the separation of luminal and basal compartments, and it was shown that the basal compartment was enriched in stem cell-like activity (Martignani et al. 2010). Sca-1 belongs to the group of surface markers which have been linked with the stemness of cells (Welm et al. 2002; Deugnier et al. 2006). In our previous study (Motyl et al. 2011), we found that only a small percentage of cells (2.14 %) expressed Sca-1 in the mammary tissue of 2-year-old HF heifers. The Sca-1^pos^ cells did not express estrogen receptor, but showed up-regulation of genes characteristic for cells of hematopoietic origin. Since Sca-1 is expressed not only by epithelial stem/progenitor cells but also by non-differentiated cells of bone marrow origin, it cannot be used as a single marker, defining a stem cell population. Therefore, in the present study, we decided to use an additional stem/progenitor cell marker, FNDC3B, to determine the epithelial stem/progenitor cell population. FNDC3B has been recently identified by microarray analysis of LREC as a potential marker of bovine MaSC (Choudhary et al. 2010). FNDC3B-positive cells were demonstrated to have increased telomerase activity, which is a feature characteristic of stem and progenitor cells (Choudhary and Capuco 2012). Cells double-labeled with antibodies against Sca-1 and FNDC3B, which were conjugated with fluorescent dyes, were counted using scanning cytometry. It is a good method for stem/progenitor cell analysis, since it allows direct examination of the cells in the tissue, without interference of its structure. Scanning cytometry enables to relocate cells with a specific phenotype in a heterogeneous tissue structure (Godlewski et al. 2008). It also gives the possibility of imaging counted cells, which not only allows to assess the accuracy of allocating cells to specific gates, but also enables to compare gated cells between the samples. A comparison of cytograms from the mammary tissues of both examined breeds revealed a significantly higher number of Sca-1^pos^FNDC3B^pos^ cells in the glandular tissue of HF than in LM heifers. We assume that the difference in the number of these cells, which can constitute a putative population of stem/progenitor cells, can belong to the factors affecting the different ability of outgrowth and development of the mammary gland, and, as a consequence, may reflect the differences in milk production between dairy and beef breed cattle.

### Identification of genes potentially regulating mammary stem cell niche in the mammary gland of heifers

**Fig. 1:**
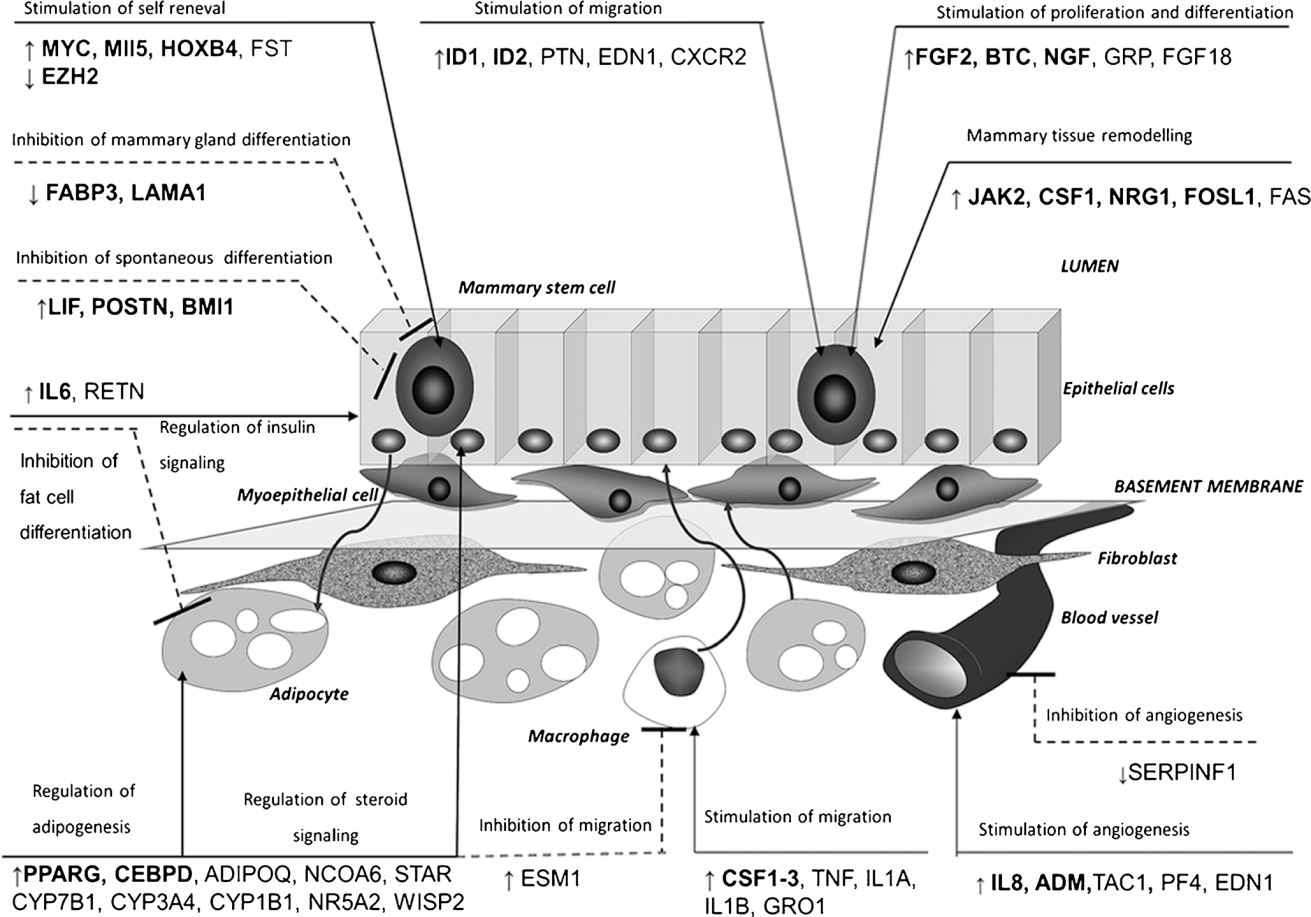
Proposed scheme of molecular regulation of the mammary stem cell microenvironment. The scheme illustrates specific dynamic regulations leading to increased potential of milk production. The stem cell niche in the mammary gland comprises mammary stem cells, as well as epithelial and myoepithelial cells, which are separated by basement membrane from adipocytes, fibroblasts, and blood vessels. The scheme was developed based on the comparison of transcriptomic profiles of mammary glands from Holstein-Friesian (HF) heifers and Limousin (LM) heifers. The names of genes regulating stem cell activity are shown in **bold**. The *arrows* indicate the direction of gene expression in dairy heifers in relation to beef heifers

Transcriptomic analyses carried out as a part of this study identified a number of biological processes, molecular functions, and signaling pathways which may pose a dairy potential in HF heifers compared to LM heifers. The literature data show that the entire functional outgrowth of mammary epithelium may comprise the progeny from a single cell (Kordon and Smith 1998). In most tissues of adult organisms, microenvironments, also called niches, are present and serve as regions of stem cell activity (LaBarge et al. 2007). Niches protect the stem cells from their excessive expansion, regulate their activity, and keep them in maintenance by homeostasis caused by the surrounding tissues (Bussard and Smith 2011). All niche structures (basement membrane, extracellular matrix) and cells of the mammary gland (progenitor, myoepithelial, fibroblasts, adipocytes, and cells of the immune system) are maintained in a state of dynamic equilibrium by adjusting auto- and paracrine regulators, including growth factors, hormones, and cytokines activity (McCave et al. 2010). The dominance of the microenvironment over the stem cell’s autonomous phenotype has been demonstrated in several reports involving cells crossing lineage “boundaries” to regenerate “foreign” tissues (Booth et al. 2008). Some very interesting data were provided by Boulanger and coworkers (2007), who observed cooperation of cells isolated from the seminiferous tubules of the mature testis into the murine mammary fat pads, contributing robust numbers of epithelial progeny to normally growing mammary glands. The same group of researchers transplanted neuronal stem cells (NSCs) from WAP-Cre/Rosa26R mice together with wild-type mammary epithelial cells into epithelium-divested mammary fat pads of prepubertal female mice, and observed that NSCs interact with MECs on transplantation, and contributed mammary epithelial-specific progeny to normal mammary outgrowths (Booth et al. 2008). The complex interactions between mammary stem/progenitor cells, stromal cells, and other components of the glandular environment are still poorly understood. However, based on the results obtained in our microarray experiment, ontological analysis, genes interaction networks, and the available literature, we have proposed a model of the mammary gland niche controlling stem cells activity (Fig. 1). The microarray data contained a number of genes associated with the activity of the MaSC and HSCs (Table 1). We revealed changes in the expression of genes involved in the maintenance, differentiation, and renewal of stem cells (Table 2).

The higher degree of development of the mammary gland in HF heifers was accompanied by the up-regulation of many genes representing factors related to stem cell maintenance and mammary tissue remodeling (Table 1). Among the up-regulated genes, we identified those encoding: Janus kinase 2 (JAK2), responsible for the regulation of alveolar cells differentiation and their maintenance during differentiation (Wagner et al. 2004); colony-stimulating factors: CSF1, CSF2, associated with the regulation of MaSC and macrophages activity, as well as stimulation of the outgrowth potential and regenerative abilities of the mammary gland (Gyorki et al. 2009); neuregulin 1 (NGR1), involved in the promotion of growth, differentiation, and stimulation of branching morphogenesis, lobulo-alveolar budding, and milk proteins production (Yang et al. 1995); transcription factor FOSL1, that takes part in the promotion of vasculogenic and angiogenic processes in the epithelium and forming tube-like structures (Evellin et al. 2013).

Among the transcripts up-regulated in the mammary gland of HF heifers were also: fibroblast growth factor 2 (FGF2), which was shown to play an important role in the differentiation of stem cells to mesodermal lineages (Sharpe et al. 2011); betacellulin (BTC), linked with the development of a lactating-like phenotype in the mammary gland of virgin female mice (Dahlhoff et al. 2011); nerve growth factor (NGF), involved in mammary tumor stem cell metastasis, proliferation, and survival (Adriaenssens et al. 2008) (Table 1). Products of the above-mentioned genes (FGF2, BTC, and JAK2) are involved in the EGFR signaling pathway, whose activity differs significantly between the two compared cattle breeds. The EGF-EGFR-mediated signaling pathway plays a key role in mammary gland development, autophagy regulation, cell cycle progression, estrogen metabolism, proliferation, cell survival, angiogenesis, and cell migration (Lurje and Lenz 2009; Sobolewska et al. 2009). Our study also revealed a group of genes differentially expressed between HF and LM breeds, which are involved in the ID signaling pathway: NGF and ID1, ID2. ID signaling is important in the regulation of maintenance, migration, and differentiation of stem cells. Increased level of proteins taking part in this pathway may result in the loss of differentiation and gain of migration and proliferative abilities (ID1), and activation of mammary differentiation and alveologenesis during pregnancy (ID2) (Itahana et al. 2003; Dong et al. 2011).

Another group of genes exerting higher expression in the mammary tissue of HF than LM comprised factors essential for the maintenance of hematopoietic stem cells’ pools, and for mammary gland involution after lactation (LIF), as well as maintenance of cellular homeostasis, mammary epithelium growth, and stem cells activity (BMI1) (Pietersen et al. 2008; Mathieu et al. 2012). We observed a lower expression of genes encoding proteins responsible for the growth arrest of mammary epithelial cells and maintenance of mammary gland progenitor cells in the quiescent state (FABP3, LAMA1) (Bionaz and Loor 2008a; Bussard and Smith 2011) (Table 2).

Interestingly, the mammary gland of HF heifers also showed a higher expression of genes involved in the regulation of stem cells renewal. We observed changes in the expression of genes which regulate the renewal of hematopoietic stem cells (HSCs), e.g., HOXB4, MLL5, TAC1, and MYB. HSCs are particularly important in the context of their penetration into the mammary gland and their potential effect on glandular development (Niku et al. 2004). HOXB4 (showing up-regulated expression in this study) activates Myc transcription factor, which also showed a higher expression. It has been demonstrated that proteins from this family of transcription factors prevent hematopoietic stem cells from differentiating, while, at the same time, allowing their self-renewal (Stoelzle et al. 2009; Lee et al. 2013). Another identified gene, MLL5, encodes a protein (myeloid/lymphoid or mixed-lineage leukemia 5), which is a key regulator of hematopoiesis involved in terminal myeloid differentiation and in the regulation of HSCs self-renewal (Heuser et al. 2009).

This study revealed also a significantly higher expression of several cytokines and growth factors in the mammary tissue of dairy heifers (HF) when compared to beef breed heifers (LM) (Table 1). IL6 and IL8 are associated with multiple processes important for mammary gland development and function, including: mammary tissue remodeling during involution, regulation of immune response, insulin signaling pathway, negative regulation of fat cell differentiation, positive regulation of epithelial cell proliferation, and promotion of angiogenesis (Zhao et al. 2002; Sansone et al. 2007; Liu et al. 2011; Singh et al. 2013). The significant difference in the expression of genes encoding these cytokines between HF and LM indicates their important role in the regulation of development and physiological function of the mammary gland.

The results of the microarray experiment also showed highly significant differences in the expression of genes involved in adipogenesis and adipose tissue activity between the examined breeds. In LM mammary glands, a higher expression of genes involved in fatty acid metabolic process, lipid storage, and lipid biosynthesis was observed. Adipocytes are essential for the development of the mammary gland. It has been shown that mammary epithelium lacking paracrine signals from stromal adipocytes forms rudimentary glandular structures with no signs of ductal branching (Landskroner-Eiger et al. 2010). Moreover, it has been suggested that white adipocytes can transdifferentiate into alveolar epithelial cell during pregnancy, whereas the alveolar epithelial cells can transdifferentiate into white adipocytes during mammary gland involution (Morroni et al. 2004). These results provide evidence that adipocyte-to-epithelium transdifferentiation constitutes another mechanism contributing to mammary gland development during pregnancy and lactation.

It is worth noting that our microarray analysis revealed a higher expression of genes involved in the oxidation/reduction process in LM than HF. The production of reactive oxygen species increases during adipogenic differentiation (Reid et al. 2013; Ogasawara et al. 2009). Differences between HF and LM in the expression of genes involved in redox balance are probably related to the fact that beef heifers have more developed mammary gland adipose tissue. It should be noted that the metabolism of beef cattle is generally more directed towards fat deposition and intramuscular lipid synthesis. The results obtained may also indicate an important effect of fatty tissue and lipid metabolism on dairy potential, but this topic is still poorly understood. The literature data indicate a relationship between adiposity and dairy potential, since fat accumulation and increased growth rate, caused by high feeding level before puberty, can lead to reduced pubertal mammary growth and reduced milk yield potential (Sejrsen et al. 2000). We revealed a higher expression of transcription regulator peroxisome proliferator-activated receptor gamma (PPARG) in HF, which plays an important role in fatty tissue activity and has many regulatory interactions with other significantly regulated genes and processes. Other reports have demonstrated that the expression of the PPARG gene in the mammary gland is correlated with the abundance of adipocytes at several stages of pregnancy, i.e., smaller number of adipocytes in late pregnancy results in lower PPARG expression (Bionaz and Loor 2008b). During lactation, the expression of PPARG is up-regulated, suggesting an important role of this nuclear receptor in milk fat synthesis (Bionaz and Loor 2008b; Kadegowda et al. 2009). PPARG was shown to regulate the expression of genes involved in triacylglyceride synthesis (LPIN1), fatty acids synthesis (ACACA, FASN, SREBF1), metabolism (SCD), and import (CD36) in bovine mammary epithelial cells (Kadegowda et al. 2009). Furthermore, several studies have shown that PPARG regulates signaling pathways, controlling and improving insulin sensitivity, cell proliferation, fatty acid β-oxidation, glucose utilization, adipocytes differentiation, and improves HSC maintenance (Ito et al. 2012; Yuan et al. 2012).

## Conclusions

In conclusion, we assume that the higher mammogenic potential in postpubertal dairy heifers in comparison with beef heifers not only depends on central endocrine regulations but also on local intramammary factors, including higher mammary stem cells (MaSC) number and auto- and paracrine regulations of the MaSC environment, forming a favorable niche for their maintenance, self-renewal, and development.

## References

[CR1] Adriaenssens E, Vanhecke E, Saule P, Mougel A, Page A, Romon R, Nurcombe V, Le Bourhis X, Hondermarck H (2008). Nerve growth factor is a potential therapeutic target in breast cancer. Cancer Res.

[CR2] Akers RM, Capuco AV, Keys JE (2006). Mammary histology and alveolar cell differentiation during late gestation and early lactation in mammary tissue of beef and dairy heifers. Livest Sci.

[CR3] Bionaz M, Loor JJ (2008). ACSL1, AGPAT6, FABP3, LPIN1, and SLC27A6 are the most abundant isoforms in bovine mammary tissue and their expression is affected by stage of lactation. J Nutr.

[CR4] Bionaz M, Loor JJ (2008). Gene networks driving bovine milk fat synthesis during the lactation cycle. BMC Genomics.

[CR5] Booth BW, Mack DL, Androutsellis-Theotokis A, McKay RD, Boulanger CA, Smith GH (2008). The mammary microenvironment alters the differentiation repertoire of neural stem cells. Proc Natl Acad Sci U S A.

[CR6] Boulanger CA, Mack DL, Booth BW, Smith GH (2007). Interaction with the mammary microenvironment redirects spermatogenic cell fate in vivo. Proc Natl Acad Sci U S A.

[CR7] Bussard KM, Smith GH (2011). The mammary gland microenvironment directs progenitor cell fate in vivo. Int J Cell Biol.

[CR8] Capuco AV (2007). Identification of putative bovine mammary epithelial stem cells by their retention of labeled DNA strands. Exp Biol Med (Maywood).

[CR9] Capuco AV, Evock-Clover CM, Minuti A, Wood DL (2009). In vivo expansion of the mammary stem/progenitor cell population by xanthosine infusion. Exp Biol Med (Maywood).

[CR10] Choudhary RK, Capuco AV (2012). In vitro expansion of the mammary stem/progenitor cell population by xanthosine treatment. BMC Cell Biol.

[CR11] Choudhary RK, Li RW, Evock-Clover CM, Capuco AV (2010). Bovine mammary stem cells: transcriptome profiling and the stem cell niche. J Dairy Sci.

[CR12] Dahlhoff M, Blutke A, Wanke R, Wolf E, Schneider MR (2011). In vivo evidence for epidermal growth factor receptor (EGFR)-mediated release of prolactin from the pituitary gland. J Biol Chem.

[CR13] Daniel CW, Smith GH (1999). The mammary gland: a model for development. J Mammary Gland Biol Neoplasia.

[CR14] Deugnier MA, Faraldo MM, Teulière J, Thiery JP, Medina D, Glukhova MA (2006). Isolation of mouse mammary epithelial progenitor cells with basal characteristics from the Comma-Dbeta cell line. Dev Biol.

[CR15] Dong J, Huang S, Caikovski M, Ji S, McGrath A, Custorio MG, Creighton CJ, Maliakkal P, Bogoslovskaia E, Du Z, Zhang X, Lewis MT, Sablitzky F, Brisken C, Li Y (2011). ID4 regulates mammary gland development by suppressing p38MAPK activity. Development.

[CR16] Eirew P, Stingl J, Raouf A, Turashvili G, Aparicio S, Emerman JT, Eaves CJ (2008). A method for quantifying normal human mammary epithelial stem cells with in vivo regenerative ability. Nat Med.

[CR17] Ellis S, Akers RM, Capuco AV, Safayi S (2012). Triennial Lactation Symposium: Bovine mammary epithelial cell lineages and parenchymal development. J Anim Sci.

[CR18] Evellin S, Galvagni F, Zippo A, Neri F, Orlandini M, Incarnato D, Dettori D, Neubauer S, Kessler H, Wagner EF, Oliviero S (2013). FOSL1 controls the assembly of endothelial cells into capillary tubes by direct repression of alphav and beta3 integrin transcription. Mol Cell Biol.

[CR19] Godlewski MM, Turowska A, Jedynak P, Martinez Puig D, Nevalainen H, Goldys EM (2008). Quantitative analysis of fluorescent image: from descriptive to computational microscopy. Fluorescence applications in biotechnology and life sciences.

[CR20] Gyorki DE, Asselin-Labat ML, van Rooijen N, Lindeman GJ, Visvader JE (2009). Resident macrophages influence stem cell activity in the mammary gland. Breast Cancer Res.

[CR21] Han J, Cao S, Jin H, Liu Y, Wang M, Song J, Li N (2006). Localization of putative stem cells and four cell populations with different differentiation degree in mouse mammary anlagen. Histochem Cell Biol.

[CR22] Heuser M, Yap DB, Leung M, de Algara TR, Tafech A, McKinney S, Dixon J, Thresher R, Colledge B, Carlton M, Humphries RK, Aparicio SA (2009). Loss of MLL5 results in pleiotropic hematopoietic defects, reduced neutrophil immune function, and extreme sensitivity to DNA demethylation. Blood.

[CR23] Hovey RC, McFadden TB, Akers RM (1999). Regulation of mammary gland growth and morphogenesis by the mammary fat pad: a species comparison. J Mammary Gland Biol Neoplasia.

[CR24] Itahana Y, Singh J, Sumida T, Coppe JP, Parrinello S, Bennington JL, Desprez PY (2003). Role of Id-2 in the maintenance of a differentiated and noninvasive phenotype in breast cancer cells. Cancer Res.

[CR25] Ito K, Carracedo A, Weiss D, Arai F, Ala U, Avigan DE, Schafer ZT, Evans RM, Suda T, Lee CH, Pandolfi PP (2012). A PML-PPAR-delta pathway for fatty acid oxidation regulates hematopoietic stem cell maintenance. Nat Med.

[CR26] Kadegowda AK, Bionaz M, Piperova LS, Erdman RA, Loor JJ (2009). Peroxisome proliferator-activated receptor-gamma activation and long-chain fatty acids alter lipogenic gene networks in bovine mammary epithelial cells to various extents. J Dairy Sci.

[CR27] Kendziorski CM, Zhang Y, Lan H, Attie AD (2003). The efficiency of pooling mRNA in microarray experiments. Biostatistics.

[CR28] Keys JE, Capuco AV, Akers RM, Djiane J (1989). Comparative study of mammary gland development and differentiation between beef and dairy heifers. Domest Anim Endocrinol.

[CR29] Kordon EC, Smith GH (1998). An entire functional mammary gland may comprise the progeny from a single cell. Development.

[CR30] LaBarge MA, Petersen OW, Bissell MJ (2007). Of microenvironments and mammary stem cells. Stem Cell Rev.

[CR31] Landskroner-Eiger S, Park J, Israel D, Pollard JW, Scherer PE (2010). Morphogenesis of the developing mammary gland: stage-dependent impact of adipocytes. Dev Biol.

[CR32] Lee J, Shieh JH, Zhang J, Liu L, Zhang Y, Eom JY, Morrone G, Moore MA, Zhou P (2013). Improved ex vivo expansion of adult hematopoietic stem cells by overcoming CUL4-mediated degradation of HOXB4. Blood.

[CR33] Liu S, Ginestier C, Ou SJ, Clouthier SG, Patel SH, Monville F, Korkaya H, Heath A, Dutcher J, Kleer CG, Jung Y, Dontu G, Taichman R, Wicha MS (2011). Breast cancer stem cells are regulated by mesenchymal stem cells through cytokine networks. Cancer Res.

[CR34] Lurje G, Lenz HJ (2009). EGFR signaling and drug discovery. Oncology.

[CR36] Martignani E, Eirew P, Accornero P, Eaves CJ, Baratta M (2010). Human milk protein production in xenografts of genetically engineered bovine mammary epithelial stem cells. PLoS One.

[CR35] Mathieu ME, Saucourt C, Mournetas V, Gauthereau X, Thézé N, Praloran V, Thiébaud P, Bœuf H (2012). LIF-dependent signaling: new pieces in the Lego. Stem Cell Rev.

[CR37] McCave EJ, Cass CA, Burg KJ, Booth BW (2010). The normal microenvironment directs mammary gland development. J Mammary Gland Biol Neoplasia.

[CR38] Morroni M, Giordano A, Zingaretti MC, Boiani R, De Matteis R, Kahn BB, Nisoli E, Tonello C, Pisoschi C, Luchetti MM, Marelli M, Cinti S (2004). Reversible transdifferentiation of secretory epithelial cells into adipocytes in the mammary gland. Proc Natl Acad Sci U S A.

[CR39] Motyl T, Bierła JB, Kozłowski M, Gajewska M, Gajkowska B, Koronkiewicz M (2011). Identification, quantification and transcriptional profile of potential stem cells in bovine mammary gland. Livest Sci.

[CR40] Niku M, Ilmonen L, Pessa-Morikawa T, Iivanainen A (2004). Limited contribution of circulating cells to the development and maintenance of nonhematopoietic bovine tissues. Stem Cells.

[CR41] Ogasawara J, Kitadate K, Nishioka H, Fujii H, Sakurai T, Kizaki T, Izawa T, Ishida H, Ohno H (2009). Oligonol, a new lychee fruit-derived low-molecular form of polyphenol, enhances lipolysis in primary rat adipocytes through activation of the ERK1/2 pathway. Phytother Res.

[CR42] Pietersen AM, Evers B, Prasad AA, Tanger E, Cornelissen-Steijger P, Jonkers J, van Lohuizen M (2008). Bmi1 regulates stem cells and proliferation and differentiation of committed cells in mammary epithelium. Curr Biol.

[CR43] Rauner G, Barash I (2012). Cell hierarchy and lineage commitment in the bovine mammary gland. PLoS One.

[CR44] Reid B, Afzal JM, McCartney AM, Abraham MR, O’Rourke B, Elisseeff JH (2013). Enhanced tissue production through redox control in stem cell-laden hydrogels. Tissue Eng Part A.

[CR45] Sansone P, Storci G, Tavolari S, Guarnieri T, Giovannini C, Taffurelli M, Ceccarelli C, Santini D, Paterini P, Marcu KB, Chieco P, Bonafè M (2007). IL-6 triggers malignant features in mammospheres from human ductal breast carcinoma and normal mammary gland. J Clin Invest.

[CR46] Sejrsen K (1994). Relationships between nutrition, puberty and mammary development in cattle. Proc Nutr Soc.

[CR47] Sejrsen K, Purup S, Vestergaard M, Foldager J (2000). High body weight gain and reduced bovine mammary growth: physiological basis and implications for milk yield potential. Domest Anim Endocrinol.

[CR48] Shackleton M, Vaillant F, Simpson KJ, Stingl J, Smyth GK, Asselin-Labat ML, Wu L, Lindeman GJ, Visvader JE (2006). Generation of a functional mammary gland from a single stem cell. Nature.

[CR49] Sharpe R, Pearson A, Herrera-Abreu MT, Johnson D, Mackay A, Welti JC, Natrajan R, Reynolds AR, Reis-Filho JS, Ashworth A, Turner NC (2011). FGFR signaling promotes the growth of triple-negative and basal-like breast cancer cell lines both in vitro and in vivo. Clin Cancer Res.

[CR50] Sheffield LG (1988). Organization and growth of mammary epithelia in the mammary gland fat pad. J Dairy Sci.

[CR51] Singh JK, Farnie G, Bundred NJ, Simões BM, Shergill A, Landberg G, Howell SJ, Clarke RB (2013). Targeting CXCR1/2 significantly reduces breast cancer stem cell activity and increases the efficacy of inhibiting HER2 via HER2-dependent and -independent mechanisms. Clin Cancer Res.

[CR52] Sleeman KE, Kendrick H, Ashworth A, Isacke CM, Smalley MJ (2006). CD24 staining of mouse mammary gland cells defines luminal epithelial, myoepithelial/basal and non-epithelial cells. Breast Cancer Res.

[CR53] Sobolewska A, Gajewska M, Zarzyńska J, Gajkowska B, Motyl T (2009). IGF-I, EGF, and sex steroids regulate autophagy in bovine mammary epithelial cells via the mTOR pathway. Eur J Cell Biol.

[CR54] Stingl J (2009). Detection and analysis of mammary gland stem cells. J Pathol.

[CR55] Stingl J, Eirew P, Ricketson I, Shackleton M, Vaillant F, Choi D, Li HI, Eaves CJ (2006). Purification and unique properties of mammary epithelial stem cells. Nature.

[CR56] Stoelzle T, Schwarb P, Trumpp A, Hynes NE (2009). c-Myc affects mRNA translation, cell proliferation and progenitor cell function in the mammary gland. BMC Biol.

[CR57] Villadsen R, Fridriksdottir AJ, Rønnov-Jessen L, Gudjonsson T, Rank F, LaBarge MA, Bissell MJ, Petersen OW (2007). Evidence for a stem cell hierarchy in the adult human breast. J Cell Biol.

[CR58] Wagner KU, Krempler A, Triplett AA, Qi Y, George NM, Zhu J, Rui H (2004). Impaired alveologenesis and maintenance of secretory mammary epithelial cells in Jak2 conditional knockout mice. Mol Cell Biol.

[CR59] Welm BE, Tepera SB, Venezia T, Graubert TA, Rosen JM, Goodell MA (2002). Sca-1(pos) cells in the mouse mammary gland represent an enriched progenitor cell population. Dev Biol.

[CR60] Yang Y, Spitzer E, Meyer D, Sachs M, Niemann C, Hartmann G, Weidner KM, Birchmeier C, Birchmeier W (1995). Sequential requirement of hepatocyte growth factor and neuregulin in the morphogenesis and differentiation of the mammary gland. J Cell Biol.

[CR61] Yuan H, Upadhyay G, Yin Y, Kopelovich L, Glazer RI (2012). Stem cell antigen-1 deficiency enhances the chemopreventive effect of peroxisome proliferator-activated receptorgamma activation. Cancer Prev Res (Phila).

[CR62] Zhao L, Melenhorst JJ, Hennighausen L (2002). Loss of interleukin 6 results in delayed mammary gland involution: a possible role for mitogen-activated protein kinase and not signal transducer and activator of transcription 3. Mol Endocrinol.

